# Spatial distribution and determinants of an optimal ANC visit among pregnant women in Ethiopia: further analysis of 2016 Ethiopia demographic health survey

**DOI:** 10.1186/s12884-020-2795-4

**Published:** 2020-03-04

**Authors:** Zemenu Tadesse Tessema, Yaregal Animut

**Affiliations:** Department of Epidemiology and Biostatistics, Institute of Public Health, College of Medicine and Health Sciences, University of Gondar, Gondar, Ethiopia

**Keywords:** Optimal antenatal care visit, Determinant, Spatial distribution, Ethiopia

## Abstract

**Background:**

Antenatal care (ANC) is essential to improve maternal and newborn health and wellbeing. Antenatal care coverage is improving in Africa since over two-thirds of pregnant women have at least one ANC contact. However, to realize the complete life-saving potential that ANC guarantees for mothers and babies, at least four visits providing essential evidence-based interventions are required.. Therefore, this study was conducted to identify determinants of an optimal ANC visit and its spatial distribution in Ethiopia.

**Methods:**

This study is a secondary data analysis of the 2016 Ethiopian Demographic and Health Survey (EDHS). A total of 8025 women who had a live birth in the five years preceding the survey were included in this study. STATA 14 software and ArcGIS10.7 software were used for analysis. The generalized estimating equation (GEE) model was fitted to identify factors associated with an optimal ANC visit. Crude and Adjusted odds ratio with a 95% CI computed to assess the strength of association between explanatory and outcome variables.

**Results:**

This study revealed that rural residence (AOR = 0.59, 95%CI: 0.45–0.77),male partners educational status [secondary school (AOR = 1.33, 95%CI: 1.05–1.67)], distance to the health institutions [not a big problem (AOR = 1.21, 95%CI: 1.04–1.39)], community-level literacy (AOR = 1.07, 95%CI: 1.03–1.12), and community level service utilization (AOR = 2.67,95%CI:2.21–3.24) were significantly associated with optimal ANC visits. From the spatial analysis result, an Optimal ANC visit was observed in Addis Ababa, Tigray, Harari, and Dire Dawa regions whereas areas with no optimal ANC visit were Afar, Amhara, Oromia Benishangul, SNNP, and Somalia regions.

**Conclusion:**

Living in peripheral regions of the country and in rural areas, lower educational status of male partners and distance to health institutions were prohibiting factors for an adequate number of visits. In this study, community-level literacy and community level service utilizations were were also affect womens’ ANC utilization which implies community-level interventions should be considered for improving antenatal care utilization and better health outcomes. The government should give special attention to the regions like Afar, Amhara, Oromia, Benishangul, SNNP, and Somalia which had low optimal ANC visits.

## Background

An estimated 303,000 women around the world died due to complications of pregnancy and childbirth in 2015. The risk of death is disproportionately high among women living in sub-Saharan Africa, yet most maternal deaths suffered each year are preventable [1]. Antenatal care utilization has acknowledged benefits in reducing maternal and fetal mortality by delivering effective and appropriate screening, preventive, or treatment interventions [2, 3]. ANC services in low-income and middle-income countries result in significant improvement in birth outcomes and long-term reductions of child mortality and malnourishment [4].

WHO recommends eight ANC contacts, although many African countries including Ethiopia are still struggling to achieve high coverage of four ANC visits [5]. Ethiopia is still using the previous recommendation of 4 ANC visit to declare a woman for having an optimal ANC visit. Each visit should consist of care that is necessary to the overall condition and stage of pregnancy and should include four main categories of care that include: identification of pre-existing health conditions, early detection of complications arising during pregnancy, health promotion and disease prevention, and birth preparedness and complication planning [6].

Other evidence also showed that attending three or fewer ANC visits in uncomplicated pregnancies is associated with increased perinatal mortality in low- and middle-income countries as compared to the recommended number of visits [7, 8]. However, only half of women worldwide receive the recommended amount of care during pregnancy. Overall 86% of pregnant women access antenatal care with skilled health personnel at least once, only three in five (62%) receive at least four antenatal visits. In places with the highest rates of maternal mortality, such as sub-Saharan Africa and South Asia, even fewer women received at least four antenatal visits (52 and 46%, respectively) [9].

Despite many efforts of the government high maternal mortality (412/100000 live births) was reported in the 2016 Ethiopian Demographic and Health Survey (EDHS). The Ethiopian government targets to reduce maternal mortality to 199/100000 live births in its Health Sector Transformation Plan (2015/16–2019/20) and one of the strategies is achieving 95% ANC utilization of at least 4 visits. Only 32% of pregnant women had 4 ANC visits with regional variations. In order to fill this gap, it is necessary to conduct researches to identify the determinants for optimal ANC visits and its spatial distribution. There is a scarcity of national data on spatial distribution and factors associated with optimal ANC visits, Therefore, the aim of this study was to assess factors associated with optimal ANC utilization and its spatial distribution using nationwide data from the Ethiopian Health and Demographic Survey 2016.

## Methods

### Study design and study settings

**Fig. 1:**
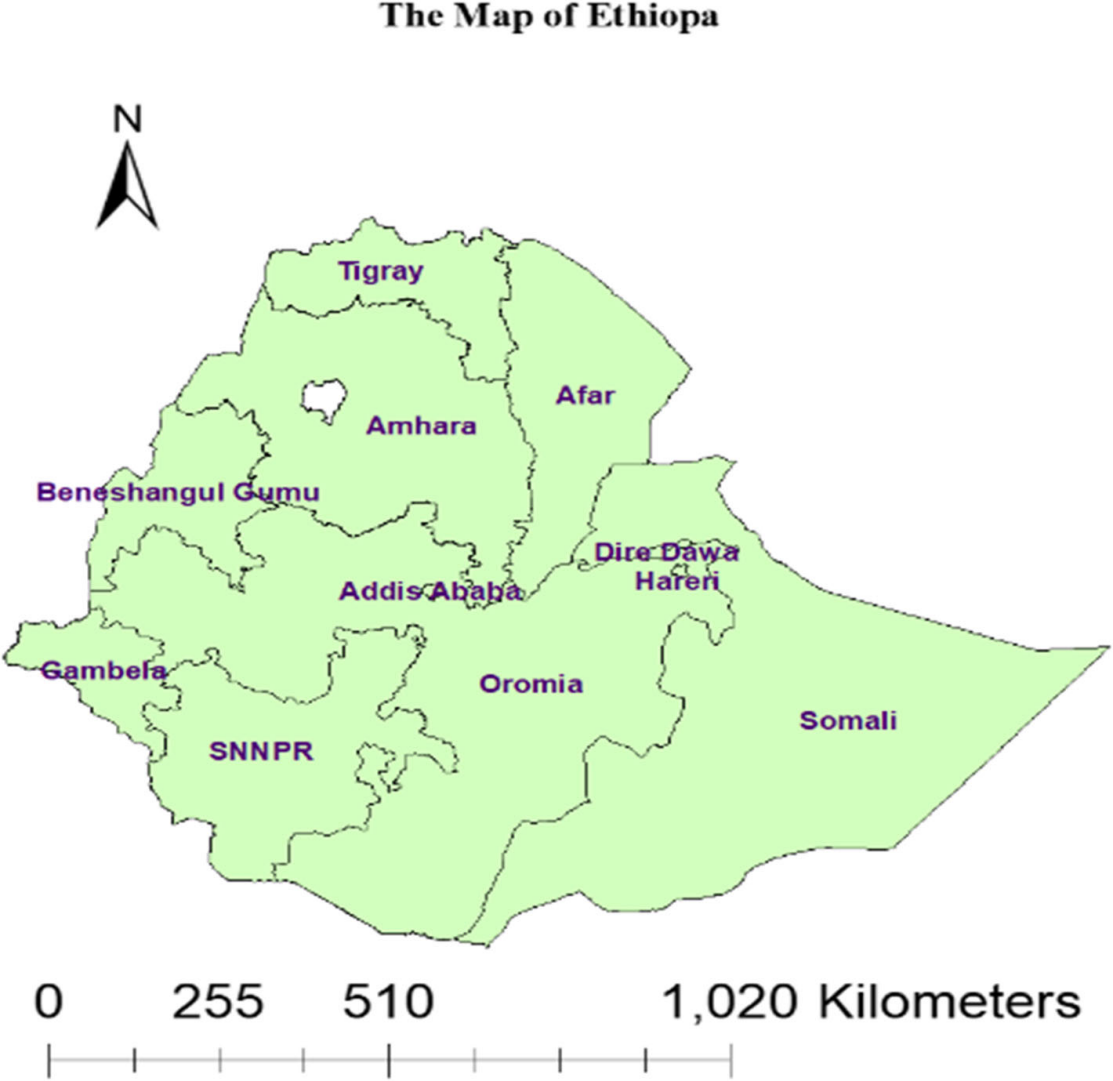
Study area of 9 regions and two city administrations in Ethiopia .(Source: Shape file from Ethiopia Central Statistical Agency (CSA), 2013 at https://africaopendata.org/dataset/ethiopia-shapefiles)

A cross-sectional study design was employed using the Ethiopian Demography and Health Surveys (EDHS) 2016. Ethiopia is located in the horn of Africa. It has a total area of 1,100,000 km2 and lies between latitudes 3° and 15°N, and longitudes 33° and 48°E. Based on the 2007 population and housing census projection, Ethiopia has a population size of 112,078,730 with 23.4% (26,226,422) of them were under reproductive age group women [10]. Ethiopia has been divided into nine ethnic-based and politically autonomous regional states (Afar, Amhara, Benishangul Gumuz, Gambela, Harari, Oromia, Somali, Southern Nations, Nationalities, and People’s Region (SNNP) and Tigray) and two cities (Addis Ababa and Dire Dawa (Fig. 1).

### Data source

This study is a secondary data analysis from the 2016 Ethiopia Demographic and Health Survey which was collected cross-sectionally from January 2015 to June 2015.

### Population and sample

All pregnant women five years preceding the survey were the study population. We used individual record (IR) files to extract the study participants of this study. The EDHS used a stratified two-stage sampling technique to select the final study participant women. Initially, the enumeration areas were stratified into urban and rural of which, 202 and 443 enumeration areas were selected from urban and rural, respectively using a probability method based on proportional to the size of Enumiration Areas (EA) and with an independent selection in each sampling stratum. In this study 622 EAs were used for spatial analysis. In EDHS 2016 a total of 16,583 eligible women were identified for individual interviews. Interviews were completed with 15,683 women, yielding a response rate of 95%.. Among 15,683 interviewed women only 8025 who had live birth in the five years preceding the survey were included in this study. For those women who had two live birth in the five years preceding the survey, only recent birth was taken. The detail of the methodology is available in the full report of 2016 EDHS [11].

### Data and variables

The Ethiopian Demographic and Health Survey (EDHS, 2016) data set was used for this analysis). The study population consists of women age 15–49 years and pregnant women five years preceding the survey. Out of 8025 women who had a live birth in the five years preceding the survey, only 2499 women had an optimal ANC visits. The binary response variable considered in this study indicated if a women completed an optimal ANC visits from skilled health care provider (Doctors, Midwives, Nurses, and Health officers) coded as 1 and 0 otherwise. Potential explanatory variables associated with completing an optimal ANC visits were based on related studies conducted on the factors influencing ANC utilization. Explanatory variables like current age, education status of women and husband, parity, marital status, sex of the household head, birth order, timing of ANC, distance to health facility and wealth index were used at individual level and residence, region, community-level ANC utilization and community level media exposure were used as community level variables.

### Spatial analysis

#### Spatial autocorrelation analysis

The spatial autocorrelation (Global Moran’s I) statistic measures whether optimal ANC utilization patterns were dispersed, clustered or randomly distributed in the study area [1]. Moran’s I is a spatial statistics used to evaluate spatial autocorrelation considering data set and produce a single output value which ranges from − 1 to + 1. Moran’s I Values close to − 1 indicate disease dispersed, whereas I close to + 1 indicate disease clustered and disease distributed randomly if I value is zero. A statistically significant Moran’s I (*p* < 0.05) leads to rejection of the null hypothesis (home delivery is randomly distributed) and indicates the presence of spatial autocorrelation.

#### Incremental autocorrelation

These peak distances are often appropriate values to use for tools with a Distance Band or Distance Radius parameter. This tool can help you select an appropriate Distance Threshold or Radius for tools that have these parameters, such as hot spot analysis [1].

#### Hot spot analysis (Getis-OrdGi* statistic)

Getis-OrdGi* statistics was computed to measure how spatial autocorrelation varies over the study location by calculating GI* statistic for each area. Z-score was computed to determine the statistical significance of clustering, and the *p*-value computed for the significance. The statistical result with high GI* indicates “hotspot” whereas low GI* means a “cold spot”.

#### Spatial scan statistical analysis

A Bernoulli-based model was used in which events at particular places were analyzed if women had an optimal ANC visit or not represented by a 1/0 variable. The scan statistics developed by Kulldorff and SaTScan™ software version 9.6 were used to identify the presence of purely spatial home delivery clusters.

#### Spatial interpolation

It is very expensive and laborious to collect reliable data in all areas of the country to know the burden of certain events. Therefore, part of a certain area can be predicted by using observed data using a method called interpolation. The spatial interpolation technique was used to predict stillbirth on the un-sampled areas in the country based on sampled EAs. There are various deterministic and geostatistical interpolation methods. Among all of the methods, ordinary Kriging and empirical Bayesian Kriging was considered the best method since it incorporates the spatial autocorrelation and it statistically optimizes the weight.. For this study, the ordinary Kriging method was used to estimate an optimal ANC visit in unsampled areas.

### Statistical analysis

#### Data processing and analysis

STATA 14.1 software used for the whole analysis of this study. Summary measures such as median with IQR, frequencies with percentages computed; tables, figures, and text used to present the results. We checked for the presence of correlation among observations within clusters (enumeration areas) and the result showed that there was a within-cluster correlation, which indicated that there is a correlation among observations at the cluster level. The generalized estimating equation (GEE) model was fitted to identify factors associated with determinants for optimal ANC visit among reproductive-age women. The generalized estimating equation was fitted with logit link function, binomial family and working correlation structures (independent, exchangeable, unstructured, and autoregressive) were compared for the smallest standard error difference of robust and model-based standard error. Finally, the exchangeable correlation was selected for this study to handle within correlation. Crude and adjusted odds ratio with a 95%CI computed to assess the strength of association between independent and outcome variable.

## Results

### Characteristics of the sample

Among the 8025 sampled women, only 31% (95% CI 29.5–33.5) had an optimal ANC visit. Out of the total women considered for this study, 69% had at least one ANC visit and 37.4% visit in third trimester. The results also showed that 7057(87%) were rural residents. Only 26.77% of rural respondents complete four or more ANC visits, while 62.95% of urban residents completed the recommended four or more ANC visits **(**Table 1).

**Table 1:** Distribution of an optimal antenatal care visit by categories of selected variables among women’s in Ethiopia, 2016

Variables	Count (%)	% of 4 or more ANC visit (optimal)
An optimal completion of ANC visit		
No	5526 (68.86)	
Yes	2499 (31.14)	100
Marital status		
Never in union	7656 (95.4)	30.88
Currently in union	369 (4.60)	36.52
Sex of household headed		
Male	6870 (85.60)	30.02
Female	1155 (14.40)	37.79
Place of Residence		
Urban	968 (12.07)	62.95
Rural	7057 (87.93)	26.77
Age		
15–19	260 (3.24)	28.60
20–24	1500 (18.69)	30.61
25–29	2503 (31.19)	32.58
30–34	1795 (22.37)	31.40
35–39	1322 (16.47)	32.01
40–44	447 (5.56)	25.45
45–49	199 (2.48)	25.02
Birth order		
1	1632 (20.33)	39.42
2–4	3399 (42.35)	32.15
> =5	2995 (37.32)	25.48
Women Level of education		
Unable to read and write	5203 (64.83)	25.71
Primary education	2198 (27.38)	36.04
Secondary education	414 (5.16)	58.04
Higher education	211 (2.63)	60.67
Husband level of education(*n* = 7578)		
Unable to read and write	3761 (49.64)	25.06
Primary education	2878 (37.98)	31.77
Secondary education	598 (7.89)	50.03
Higher education	341 (4.49)	53.04
Wealth quartile		
Poor	3649 (45.47)	22.74
middle	1613 (20.10)	29.25
rich	2763 (34.43)	43.21
Parity		
1	2145 (30.54)	38.55
2–5	3095 (38.57)	37.13
> 5	2479 (30.89)	24.32
Distance to health facility		
Big problem	4823 (60.10)	46.76
Not big problem	3202 (39.90)	54.24
Media Exposure		
Exposure	5399 (68.24)	58.03
Non Exposed	2513 (31.76)	41.97
Region		
Tigray	532 (6.63)	57.10
Afar	78 (0.97)	19.99
Amhara	1671 (20.83)	30.73
Oromia	3436 (42.82)	22.22
Somalia	322 (4.01)	10.43
Benishangul Gumuz	88 (1.10)	41.52
SNNP	1623 (20.33)	38.09
Gambela	22 (0.27)	41.53
Harari	19 (0.24)	32.54
Addis Ababa	198 (2.47)	89.08
Dire Dawa	34 (0.43)	64.32
Community level ANC		
low	2776 (32.09)	19.86
high	5450 (67.91)	55.00
Community level literacy		
low	3524 (43.91)	39.64
high	4502 (56.09)	24.48
Timing of ANC		
Less than 12 weeks	1092 (13.61)	23.29
More than 12 weeks	6933 (86.39)	76.71

### Spatial analysis results

#### Spatial autocorrelation

**Fig. 2:**
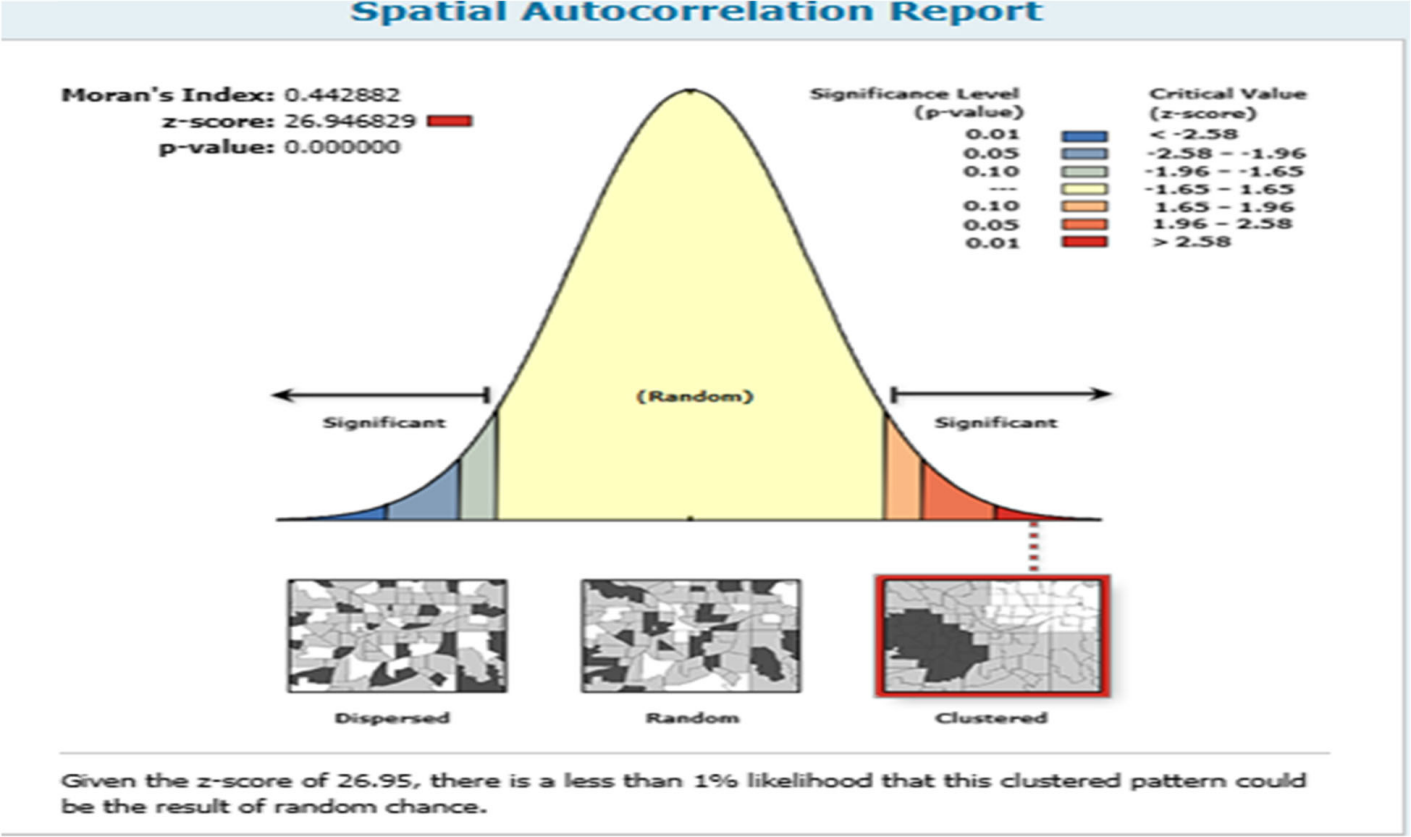
Spatial autocorrelation of an Optimal ANC visit in Ethiopia across regions (Source: Shape file from Ethiopia Central Statistical Agency (CSA), 2013https://africaopendata.org/dataset/ethiopia-shapefiles)

The spatial autocorrelaton results revealed that an optimal ANC visits had clustering effct in Ethiopia (means in some areas there was high an Optimal ANC visits and in Some areas there were low an Optimal ANC visits). The outputs have automatically generated keys on the right and left sides of each panel. The z score of 26.94 indicated that there is less than 1% likelihood that this clustered pattern could be the result of random chance. The bright red and blue colors to the end tails indicate an increased significance level. The table shows that the observed value is greater than the expected value and *P*-value is < 0.05, thus it is statistically significant and means that there is spatial variability in optimal utilization of ANC among pregnant women in Ethiopia. (Fig. 2).

#### Spatial distribution of subjects who completed the optimal number of ANC visits in Ethiopia

**Fig. 3:**
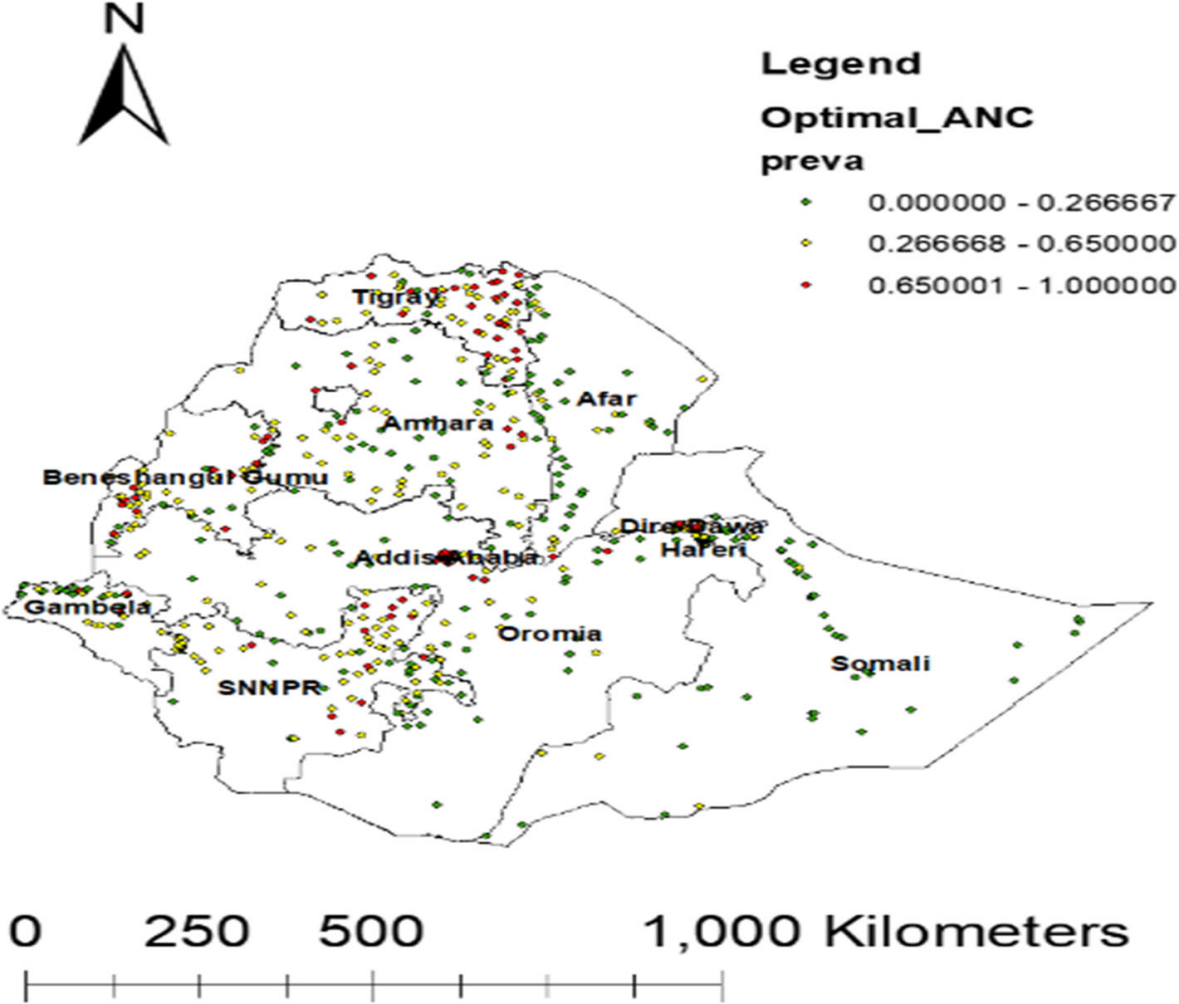
Spatial distribution of an optimal ANC visit across the (Source: Shape file from Ethiopia Central Statistical Agency (CSA), 2013 https://africaopendata.org/dataset/ethiopia-shapefiles)

A total of 622 clusters were considered for the spatial analysis of an optimal ANC visit. Each point on the map represents one enumeration area with a proportion of an Optimal ANC visit in each cluster. The red color indicates areas with a high proportion of optimal ANC whereas blue color indicates EAs with lower proportion an optimal ANC visit (Fig. 3).

#### Incremental autocorrelation

**Fig. 4:**
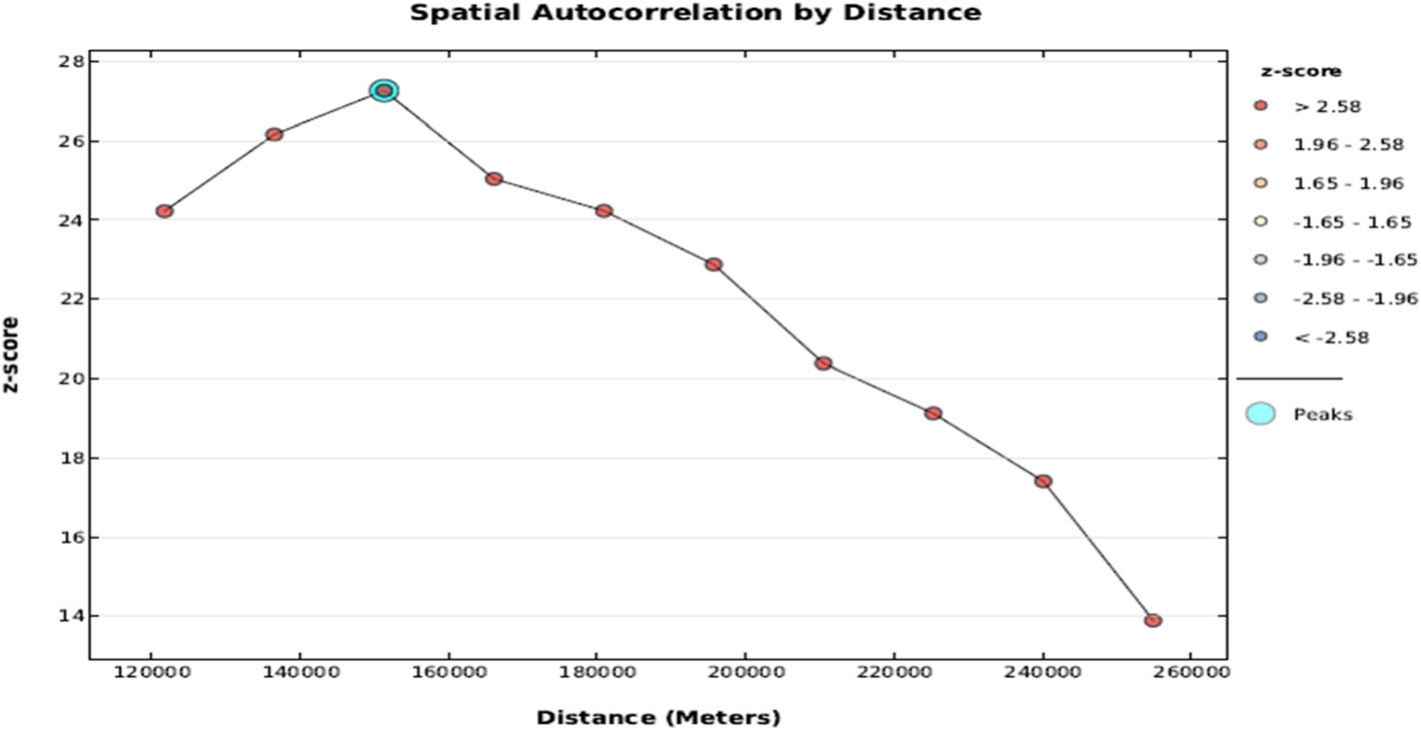
Incremental autocorrelation of optimal ANC utilization among pregnant women in Ethiopia,2016 (Source: Shape file from Ethiopia Central Statistical Agency (CSA), 2013 https://africaopendata.org/dataset/ethiopia-shapefiles)

Incremental spatial autocorrelation for a series of distance presented by a line graph. A corresponding Z-score was done to determine the average nearest neighbor, minimum, and maximum distance band. Totally 10 distance bands were detected by a beginning distance of 121,803 m, and first maximum peak (clustering) was observed at 151379.64 m (Fig. 4).

#### Hot spot analysis of optimal ANC visit in Ethiopia

**Fig. 5:**
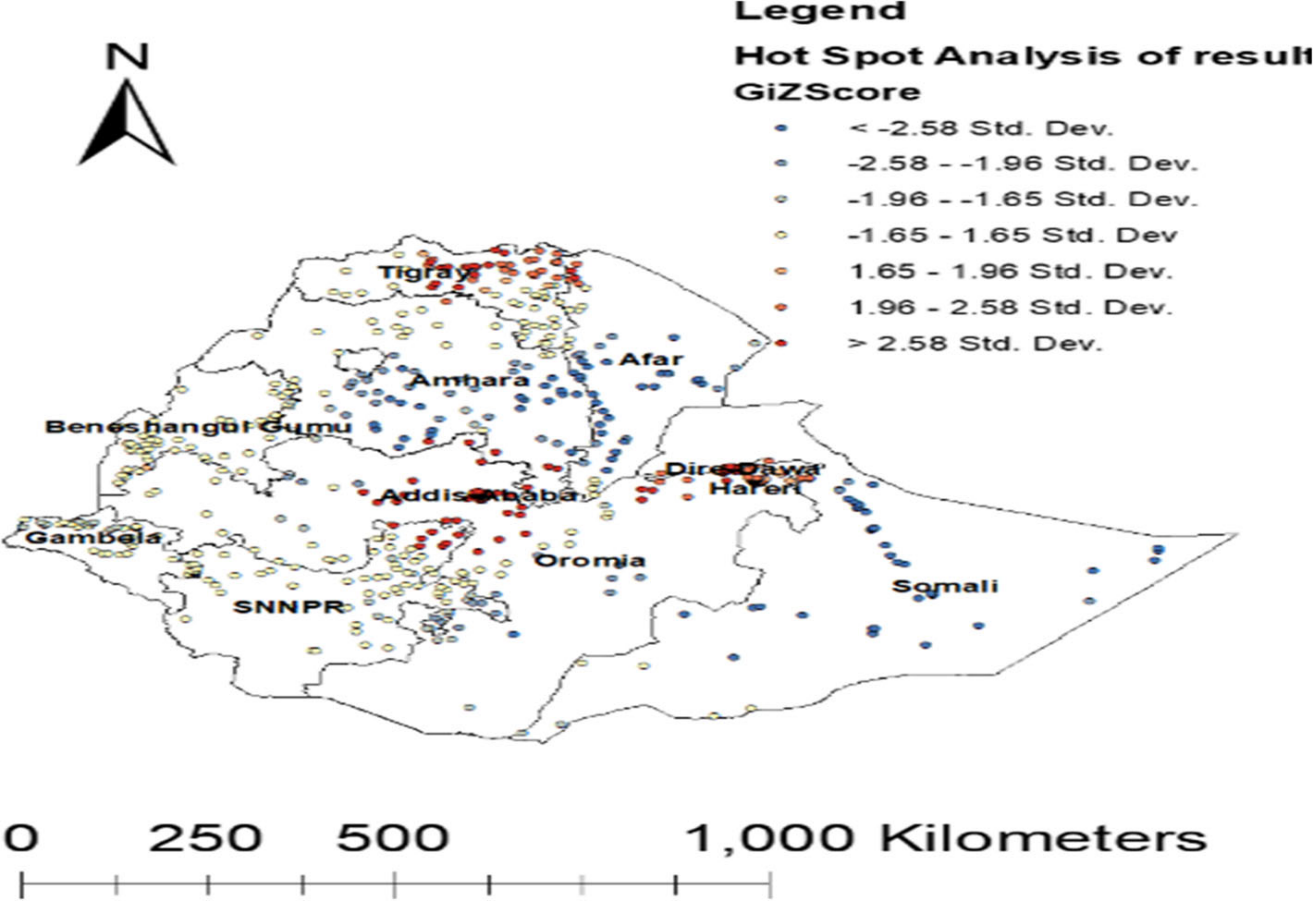
Map of Hot spot analysis of an optimal ANC utilization among reproductive-age women in Ethiopia (Source: Shape file from Ethiopia Central Statistical Agency (CSA), 2013 https://africaopendata.org/dataset/ethiopia-shapefiles)

The red color indicates that significant areas to have an optimal ANC visit. This is found in Addis Ababa, Tigray region, Harari and Diredawa whereas, the blue color indicates significant areas that had no optimal ANC visit observed in the Somalia region, Amhara region, Afar Region, Oromia region and Gambella region (Fig. 5).

#### Interpolation of an ANC visit

**Fig. 6:**
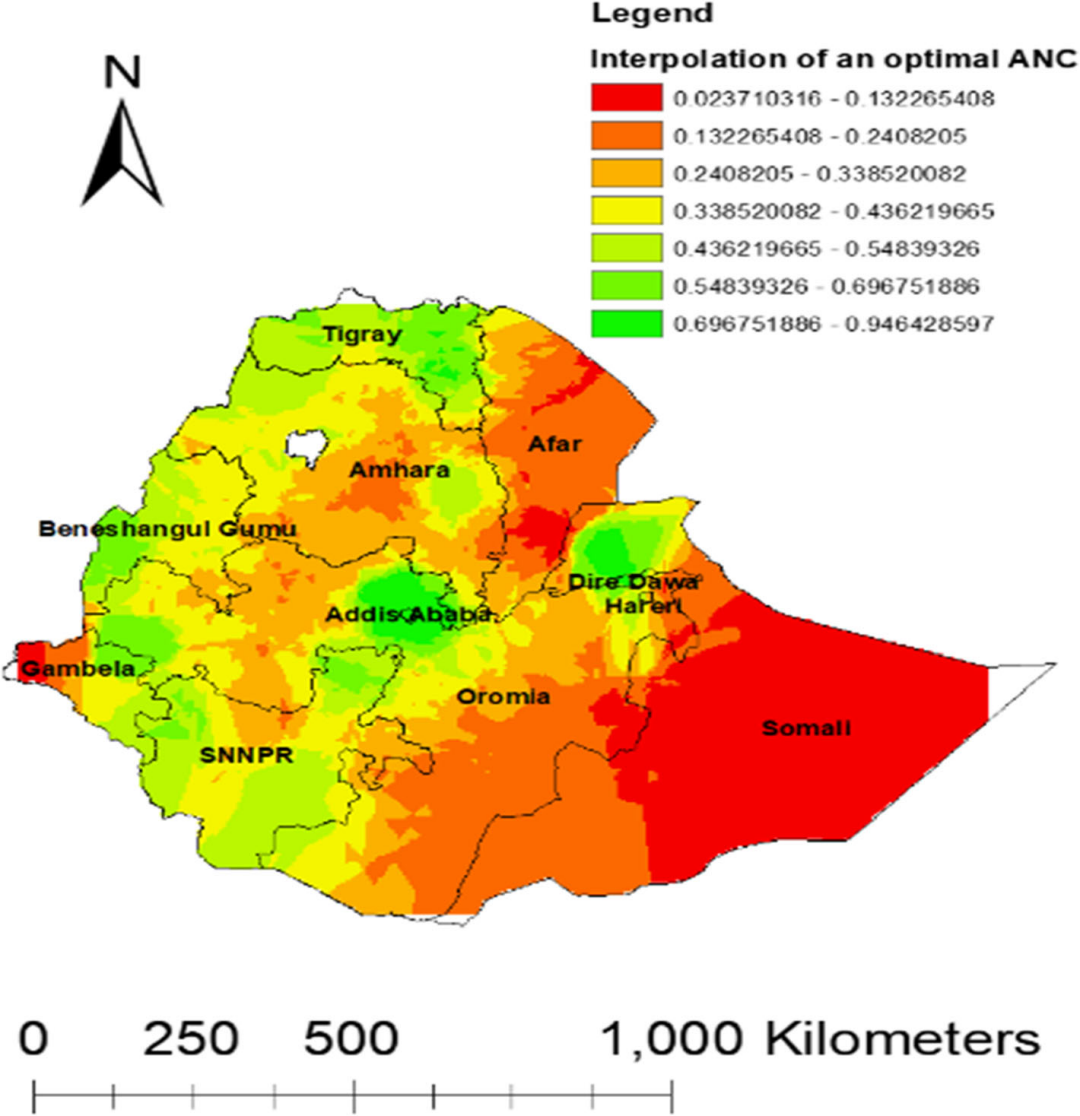
Interpolation Optimal ANC visit in Ethiopia 2016 (Source: Shape file from Ethiopia Central Statistical Agency (CSA), 2013 https://africaopendata.org/dataset/ethiopia-shapefiles)

The interpolated spatial analysis result predicted the unsampled areas. The red color indicates the predicted not had an Optimal ANC visit and the green color indicates the predicted high optimal ANC visit areas. The map indicated that Afar, Somali, and Gambella are regions which had low optimal ANC visits **(**Fig. 6).

#### Spatial SaTScan analysis of an optimal ANC visit Bernoulli based model

Most likely primary clusters (means areas who had high an Optimal ANC visits) and secondary clusters (high an Optimal ANC visits but relatively less than pimary clusters) of an optimal ANC visits were identified. About 111 (priary and secondary) significant clusters were identified. Of which, 55 of them were most likely (primary) clusters and 56 were secondary clusters.

**Fig. 7:**
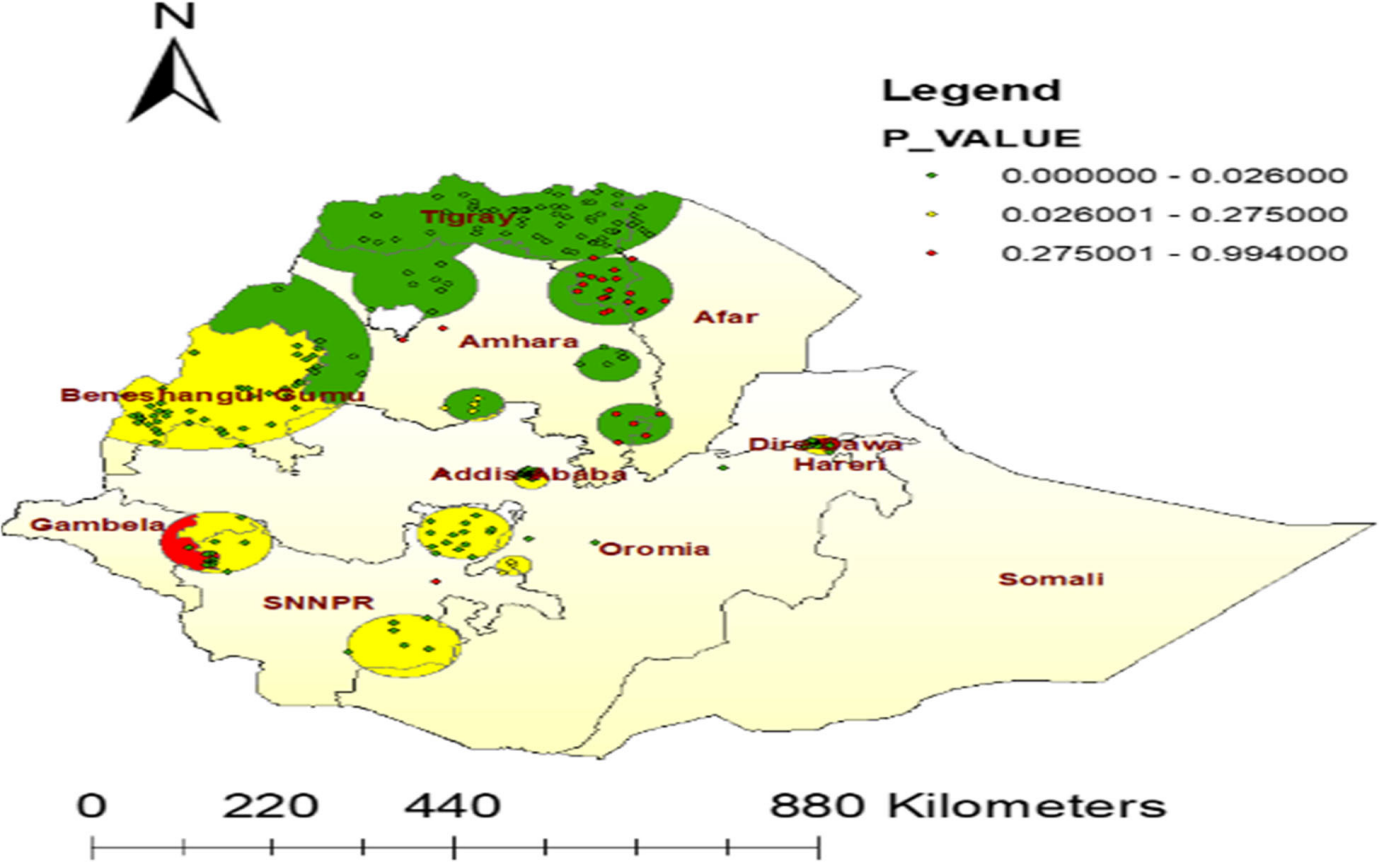
Sat Scan Statistics of An Optimal ANC visit in Ethiopia 2016 (Source: Shape file from Ethiopia Central Statistical Agency (CSA), 2013 https://africaopendata.org/dataset/ethiopia-shapefiles)

The primary cluster’s spatial window was located in the west Benishangul, which was centered at (8.883803 N, 38.778503 E) / 21.03 km, RR = 2.94 and Log-Likelihood ratio (LLR) of 145.88 at *p* < 0.001. Women who live in the primary clusters increase an optimal ANC visits by three fold than women’s outside the window (Table 2**,** Fig. 7).

**Table 2:** SaTScan analysis of An optimal ANC visit among women in the last five years in Ethiopia, 2016

Cluster	EA (enumeration Area)	Coordinate or Radi	RR	LLR	P-value
Primary(55)	236, 252, 83, 353, 475, 261, 539, 451, 61, 225, 264, 110, 302, 293, 330, 159, 19, 211, 645, 59, 608, 155, 195, 145, 487, 314, 639, 428,635, 414, 509, 560, 305, 15, 582, 147, 100, 108, 247, 31, 107, 626, 153, 170, 402, 369, 339, 91, 11, 532, 464, 144, 90, 287, 463, 112	(8.883803 N, 38.778503 E) / 21.03 km	**2.94**	**145.88**	**< 0.001**
Secondary(56)	89, 479, 45, 461, 84, 598, 404, 481, 413, 604, 81, 590, 400, 226, 597, 341, 355, 636, 103, 129, 117, 192, 156, 584, 196, 181, 430, 579,263, 623, 528, 134, 255, 99, 298, 575, 551, 98, 220, 127, 78, 362 94, 237, 550, 605, 340, 235, 538, 384, 424, 188, 583, 585, 268, 421, 160	(14.438634 N, 39.085800 E) / 143.67 km	**1.91**	**60.98**	**< 0.001**

#### Determinants for an optimal ANC visit

In the multivariate analysis, residence, religion, male partner’s educational level, distance to the health institution, region, the timing of ANC,community-level literacy and community level service utilization were significantly associated with optimal ANC visit at p-value 0.05.

The odds of optimal ANC utilization is reduced by 41% among rural women (AOR = 0.59, 95%CI: 0.45–0.77) as compared to women residing in urban areas. The odds of optimal ANC utilization is reduced by 29% for Protestants (AOR = 0.71, 95%CI: 0.55–0.91) and 48% for Catholic & traditional (AOR = 0.52, 95%CI: 0.33–0.83) as compared to Orthodox Christian followers. Women, whose partners attain the secondary level of education, are 1.33 times (AOR = 1.33, 95%CI: 1.05–1.67) more likely to have 4 ANC visits as compared to women who have a partner with no formal education. Women who reported that distance to a health institution is not a big problem are 1.21 times (AOR = 1.21, 95%CI: 1.04–1.39) more likely to have optimal ANC visits than their counterparts. Women who start ANC after 12 weeks of gestation are less likely to have adequate ANC visits (AOR = 0.70, 95%CI: 0.60–0.82) than those who start before 12 weeks of gestation. Pregnant women residing in regional states of Ethiopia are less likely to have optimal ANC visits, Tigray (AOR = 0.48, 95%CI: 0.28–0.82), Afar (AOR = 0.13, 95%CI: 0.07–0.24), Amhara (AOR = 0.24, 95%CI: 0.14–0.42), Oromia (AOR = 0.25, 95%CI: 0.15–0.43), Somali (AOR = 0.08, 95%CI: 0.05–0.15), Benishangul (AOR = 0.48, 95%CI: 0.28–0.84), Southern Nations Nationalities and Peoples Region (SNNPR) (AOR = 0.0.48, 95%CI: 0.28–0.83), Gambela (AOR = 0.37, 95%CI: 0.21–0.66), Harari (AOR = 0.16, 95%CI: 0.09–0.28), Diredawa (AOR = 0.52, 95%CI: 0.29–0.93), than women in the capital city, Addis Ababa. Women who live in a community where the distance to the health institution is not a big problem for a higher proportion of the women in the community are 1.28 times (AOR = 0.128, 95%CI: 1.04–1.57) more likely to have optimal ANC visits (Table 3).

**Table 3:** Bi-variable and multi-variable odds ratio for potential factors of completing four or more ANC visit in Ethiopia, EDHS 2016

Variables	COR(95%CI)	AOR(95%CI)
Marital status		
Never in union	ref	
Currently in union	0.99 (0.97,1.03)	0.95 (0.91,1.00)
Sex of household headed		
Male	Ref	
Female	1.02 (0.99,1.05)	1.13 (0.98,1.37)
Place of Residence		
Urban	Ref	
Rural	0.66 (0.63,0.69)	0.59 (0.45,0.77)**
Age		
15–19	Ref	Ref
20–24	1.02 (0.97,1.08)	1.07 (0.76,1.50)
25–29	1.02 (0.97,1.08)	1.18 (0.82,1.68)
30–34	1.02 (0.96,1.07)	1.23 (0.84,1.80)
35–39	1.03 (0.97,1.08)	1.32 (0.88,1.98)
40–44	0.95 (0.91,1.04)	1.01 (0.63,1.60)
45–49	0.96 (0.89,1.05)	1.02 (0.57,1.83)
Religion		
Orthodox	Ref	Ref
Muslim	0.52 (0.44,0.62)	1.03 (0.82, 1.28)
Protestant	0.60 (0.49,0.73)	0.71 (0.55,0.91)*
Catholic & traditional	0.53 (0.37,0.75)	0.52 (0.33,0.83)*
Birth order		
1	Ref	
2–4	0.96 (0.93,0.98)	0.88 (0.71,1.09)
> =5	0.93 (0.91,0.96)	0.85 (0.62,1.17)
Women Level of education		
Unable to read and write	Ref	
Primary education	1.05 (1.02,1.07)	0.96 (0.82,1.13)
Secondary education	1.16 (1.11,1.21)	1.02 (0.77,1.35)
Higher education	1.23 (1.16,1.31)	1.02 (0.67,1.56)
Parity		
1	Ref	Ref
2–5	0.80 (0.72,0.89)	0.92 (0.75,1.14)
≥ 5	0.74 (0.65,0.83)	0.94 (0.68,1.29)
Wealth quartile		
Poor	Ref	
middle	1.04 (1.01,1.07)	1.09 (0.91,1.31)
rich	1.17 (1.14,1.21)	1.10 (0.91,1.33)
Distance to the health institution		
Not a big problem	ref	ref
A big problem	1.44 (1.30,1.59)	1.21 (1.04,1.39)*
Exposure to media		
Yes	Ref	Ref
No	1.55 (1.39,1.72)	1.09 (0.94,1.28)
Partner’s level of education		
no education	Ref	ref
Primary	1.19 (1.07,1.33)	1.06 (0.92,1.22)
Secondary	1.81 (1.53,2.14)	1.33 (1.05,1.67)
Higher	1.69 (1.40,2.05)	1.02 (0.76,1.36)
Region		
Tigray	0.73 (0.67,0.79)	0.48 (0.28,0.82)*
Afar	0.48 (0.44,0.52)	0.13 (0.07,0.24)*
Amhara	0.56 (0.51,0.61)	0.24 (0.14,0.42)*
Oromia	0.52 (0.48,0.56)	0.25 (0.15,0.43)*
Somalia	0.45 (0.42,0.49)	0.08 (0.05,0.15)*
Benishangul Gumuz	0.62 (0.57,0.68)	0.48 (0.28, 0.84)*
SNNP	0.61 (0.56,0.66)	0.48 (0.28,0.83)*
Gambela	0.59 (0.54,0.64)	0.37 (0.21,0.66)*
Harari	0.60 (0.55,0.56)	0.16 (0.09,0.28)*
Addis Ababa	Ref	Ref
Dire Dawa	0.80 (0.71,0.88)	0.52 (0.29,0.93)*
Community level ANC utilization		
high	Ref	Ref
low	1.54 (1.49,1.59)	2.67 (2.21,3.24)*
Community level literacy		
low	Ref	Ref
high	1.38 (1.32,1.43)	1.07 (1.03,1.12)*
Community level media exposure		
Higher media exposure	Ref	Ref
Lower media exposure	1.50 (1.05,1.75)	1.16 (0.95,1.42)
Timing of ANC		
Less than 12 weeks	Ref	Ref
More than 12 weeks	0.80 (0.58,0.90)	0.70 (0.60,0.82)

Ref indicated reference category

*indicated significant at 5% level of significant

## Discussion

The odds of optimal ANC utilization was reduced by 41% among rural women as compared to women residing in urban areas. This finding is supported by studies done in Indonesia [12], Nigeria [13], and Ethiopia [14]. This is due to the socioeconomic inequalities and differences in health services access between urban and rural areas in the country [15].

The odds of optimal ANC utilization is reduced by 29% for Protestants and 48% for Catholic and others as compared to Orthodox Christian followers. The effect of religion on maternal health service utilization is because it plays a significant role in shaping beliefs, norms, and values including those that relate to childbirth and health services use [16–18]. Reproductive health issues may also be considered as a subject not to be discussed easily between husband and wife in some religions [19].

Women, whose partners attain a secondary level of education, were 1.33 times more likely to have 4 ANC visits as compared to women who have a partner with no formal education. Similar findings were reported in studies conducted in Bangladesh [20], Debrebirhan central Ethiopia [21] and Metekel, western Ethiopia [22]. In Ethiopia, it is known that most women are socioeconomically dependent on male partners who are decision-makers in households, and influence on maternal health care services utilization [23].

Women living in the regional states had lower odds of optimal ANC visits than women living in Addis Ababa. There was a significant difference in antenatal care utilization across the country regions. This finding is supported by a previous study conducted in Ethiopia [24]. Addis Ababa is the capital city of the country where health facilities are more accessible and women are more aware of maternal health services.

Distance to the health facilities was an important predictor for optimal ANC utilization. Women who reported distance to the health institution as not a big problem had 21% higher odds of optimal ANC visits than their counterparts. The finding is consistent with studies conducted in Indonesia [12], Uganda [18] and Tanzania [25]. These findings showed that the improvement of access to health services as well as the distribution of health services and especially in remote areas should be a priority.

This study revealed that the timing of the first ANC visit was significantly associated with the accomplishment of 4 ANC visits. Women who start their first visit after the 12 weeks of gestation are 70% less likely to have 4 ANC visits. This is due to that women with delayed initiation of ANC may deliver before getting an adequate number of ANC visits. On the other hand, women who start ANC visit early, are more likely to have better awareness about the importance of the service and committed to attained consecutive visits.

In this study community-level factors like Community level literacy rate and community level ANC utilization rate were found to be important determinants for optimal ANC visit. Women from a community where there is a higher level of literacy and a high proportion of ANC utilization are more likely to have adequate ANC visits. This may be due to the herd effect of the community level behavior. Previous studies also suggest that community-level factors could lead to an increase in the utilization of maternal health care services [26, 27].

This study has strengths of nationally representative data, advanced statistical models were used to account correlations within clusters, and spatial analysis was used to indicate hotspot areas. However, this study has limitations of cross-sectional nature that may not show a true causal relationship. In addition, the effects of the health system and health worker factors were not assessed.

## Conclusion

In this study, we have identified both individual-level and community-level factors, which determine the accomplishment of four ANC visits for pregnant women (an optimal ANC visit), and its spatial distribution. Women who lived in peripheral regions and rural areas, far from health institutions, start ANC after the 12 weeks of gestation and with a lower level of husband’s education were less likely to complete four ANC visits. Whereas, women who were in a community where there are higher-level community level literacy and community level service utilizations were more likely to have adequate ANC visits. Therefore, interventions should focus on the involvement of male partners and at the community level for improving antenatal care utilization and better health outcomes and special attention should be given to regions like Somalia, Afar, and Gambella where a proportion of an Optimal ANC visit is low.

## Data Availability

The datasets used and/or analysed during the current study are available from the corresponding author on reasonable request.

## Abbreviations

ANC: Antenatal Care Visit
AOR: Adjusted Odds Ratio
CI: Confidence Interval
EDHS: Ethiopia Demographic and Health Survey
GEE: Generalized Estimating Eqs.
IR: Individual Records
SNNP: Southern Nation and Nationalities of People of Ethiopia
